# Our Thanks

**Published:** 1871-11

**Authors:** 


### PART III.

### Editorials, Correspondence and Miscellaneous.

### OUR THANKS.

WE sincerely thank those of our Editors and friends who have forwarded us communications for our Original Department. We desire, especially, to have this department of our journal constantly filled with communications. Nothing adds more to the interest—to say nothing of the reputation—of a journal than an able corps of contributors. This, it is the good fortune of the COMPANION

to have. We hope our friends will remember this appeal, and forward articles *at once*. We promise to insert as soon as possible after receiving them. We also hope to start upon the Second Volume of THE COMPANION with an abundance of material in the shape of Communications. If our friends will respond to our efforts to serve them, we trust to make a journal which shall not only be of the greatest service to our brethren, but one which all may justly approve, endorse, and support.

Our associate, Dr. Chas. S. Lewis, of Virginia, suggests that each editor select twenty or more good men to whom shall be sent specimen numbers of THE COMPANION, with the request that each of these gentlemen act as agents. We trust our editors will, if they approve of the suggestion, at once send us the names of such gentlemen for the purpose suggested.

